# Stereotactic Magnetic Resonance-Guided Adaptive and Non-Adaptive Radiotherapy on Combination MR-Linear Accelerators: Current Practice and Future Directions

**DOI:** 10.3390/cancers15072081

**Published:** 2023-03-30

**Authors:** John Michael Bryant, Joseph Weygand, Emily Keit, Ruben Cruz-Chamorro, Maria L. Sandoval, Ibrahim M. Oraiqat, Jacqueline Andreozzi, Gage Redler, Kujtim Latifi, Vladimir Feygelman, Stephen A. Rosenberg

**Affiliations:** Department of Radiation Oncology, H. Lee Moffitt Cancer Center and Research Institute, Tampa, FL 33612, USA; john.bryant@moffitt.org (J.M.B.);

**Keywords:** radiation therapy, RT, ultra-hypofractionated radiation therapy, ablative radiation therapy, adaptive radiation therapy, image guided radiotherapy, magnetic resonance imaging, MRI, MR-guided radiation therapy, MRgRT, stereotactic body radiotherapy, SBRT, stereotactic ablative radiotherapy, SABR, stereotactic magnetic resonance-guided adaptive radiotherapy, SMART, plan optimization, tumor motion management, multiparametric MRI, mpMRI

## Abstract

**Simple Summary:**

Stereotactic body radiotherapy (SBRT) is an effective radiation therapy technique that heavily relies upon daily image guidance to achieve the necessary precision. Magnetic resonance imaging (MRI) offers significant advantages over computed tomography (CT), which has traditionally been used for daily image guidance for SBRT. Hybrid MRI and linear accelerators (MRLs) allow for the delivery of stereotactic MR-guided adaptive radiotherapy (SMART) and improve patient outcomes for many types of tumors. In this review, we summarized the evidence for SMART as it related to ablative treatments and explored how multi-parametric MRIs could continue to improve patient outcomes.

**Abstract:**

Stereotactic body radiotherapy (SBRT) is an effective radiation therapy technique that has allowed for shorter treatment courses, as compared to conventionally dosed radiation therapy. As its name implies, SBRT relies on daily image guidance to ensure that each fraction targets a tumor, instead of healthy tissue. Magnetic resonance imaging (MRI) offers improved soft-tissue visualization, allowing for better tumor and normal tissue delineation. MR-guided RT (MRgRT) has traditionally been defined by the use of offline MRI to aid in defining the RT volumes during the initial planning stages in order to ensure accurate tumor targeting while sparing critical normal tissues. However, the ViewRay MRIdian and Elekta Unity have improved upon and revolutionized the MRgRT by creating a combined MRI and linear accelerator (MRL), allowing MRgRT to incorporate online MRI in RT. MRL-based MR-guided SBRT (MRgSBRT) represents a novel solution to deliver higher doses to larger volumes of gross disease, regardless of the proximity of at-risk organs due to the (1) superior soft-tissue visualization for patient positioning, (2) real-time continuous intrafraction assessment of internal structures, and (3) daily online adaptive replanning. Stereotactic MR-guided adaptive radiation therapy (SMART) has enabled the safe delivery of ablative doses to tumors adjacent to radiosensitive tissues throughout the body. Although it is still a relatively new RT technique, SMART has demonstrated significant opportunities to improve disease control and reduce toxicity. In this review, we included the current clinical applications and the active prospective trials related to SMART. We highlighted the most impactful clinical studies at various tumor sites. In addition, we explored how MRL-based multiparametric MRI could potentially synergize with SMART to significantly change the current treatment paradigm and to improve personalized cancer care.

## 1. Introduction

Cancer continues to be a major global health concern and a leading cause of death. There were an estimated 19.3 million new cancer diagnoses and 10.0 million cancer-related deaths worldwide in 2020 [1]. By 2040, it is estimated that there will be 29.5 million new cases and 16.3 million deaths annually worldwide [2]. Radiotherapy (RT) remains a fundamental component of an effective cancer treatment program [2]. An estimated 50% of all cancer patients receive RT as part of their care [3]. Therefore, advances within the field of radiation oncology are paramount to the improvement of cancer outcomes. Stereotactic body radiotherapy (SBRT) has emerged as a highly effective RT modality that allows for radiotherapeutic-dose escalation that can be delivered in fewer fractions, as compared to conventionally dosed RT [4]. However, this new modality requires more exact targeting to ensure that these high doses are delivered to the tumor, not the healthy, tissue. SBRT has traditionally relied on planar or volumetric (e.g., cone-beam computed tomography (CBCT)) X-ray imaging techniques to ensure proper treatment planning each day to improve accuracy [5]. However, X-ray imaging techniques are insensitive to morphological changes, relative to the tumor, in the surrounding soft tissue [6], which are often the most radiosensitive and at risk of significant treatment-related toxicity [7,8,9]. CBCT has lacked the ability to accurately delineate the interface between tumor and normal soft tissue, which has limited the dose that could be safely planned for delivery [10]. Additionally, intrafraction motion management with X-ray-based imaging has often relied on a surrogate, such as an external patient surface and internal fiducial markers [11]. A recent development within the field of radiation oncology is the magnetic resonance imaging-guided linear accelerators (MRLs) that can overcome some of the challenges associated with X-ray/CT-based systems.

**Figure 1:**
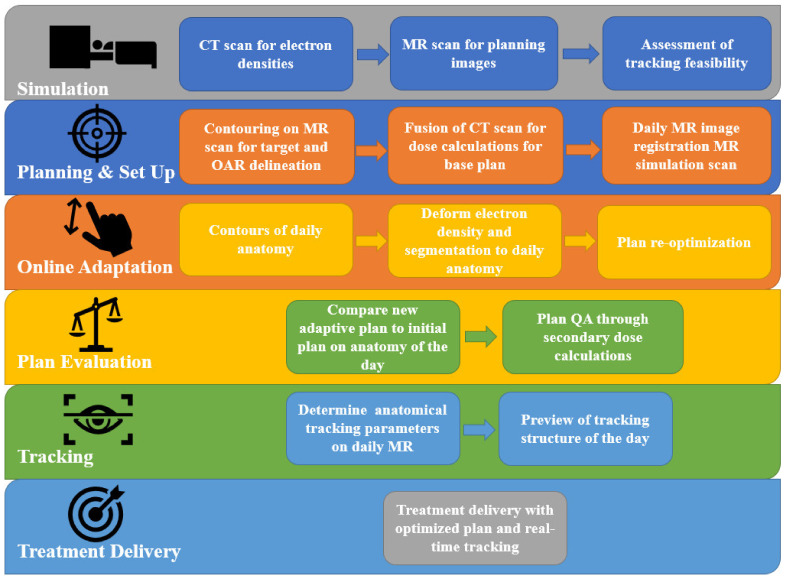
MRL workflow. CT: Computed tomography; MR: magnetic resonance; MRL: MR linear accelerator; OAR: organ at risk; QA: quality assurance.

Magnetic resonance imaging (MRI) offers improved soft-tissue delineation, allowing for the better visualization and discrimination of normal tissues and tumor targets, while being able to detect subtle physiological changes within the tissues, as well [12,13]. MR-guided RT (MRgRT) has traditionally used offline MRI to assist in defining volumes during the initial planning stages [14,15]. This contrasts with online MRgRT, which allows for daily on-table MRI and for the direct monitoring of targets and critical organs at risk (OARs) during treatment. Online MRI is the defining feature of MRL that provides all its unique capabilities and online adaptive workflow, as shown in Figure 1. MRL can acquire MR images for both treatment planning and daily set-up verification with the patient in the treatment position. Prior to treatment, each MRI acquired can be used for adjusting the plan to account for the exact positions of the targets and the normal tissue when fused with a treatment-planning CT [16,17]. When combined with dedicated software and efficient workflows, this daily MR-based adaptive planning allows for improved target coverage, opportunities for isotoxic dose delivery, and reduced normal tissue toxicity. This is called online adaptive radiotherapy and may increase the therapeutic window of RT. In addition, the MRL is capable of real-time (cine) MRI while the treatment is being delivered according to a rapid and balanced steady-state free-precession MRI acquisition technique [18], allowing for treatment-gating based on the patient anatomy directly (e.g., the tumor target) for motion control. These capabilities reduce the uncertainties in external beam radiation therapy delivery. Traditionally, larger planning target volume (PTV) margins have been used to account for these uncertainties and ensure that we treat the target appropriately. However, the unique capabilities of MRL allow for margin reduction. This, in turn, allows for higher tumor doses while conserving the normal tissue and, Therefore, widening the therapeutic window for the safe and effective delivery of MRI-guided SBRT (MRgSBRT).

This increased therapeutic window allows for safer isotoxic dose escalation. Online adaptive SBRT in an MRL is commonly referred to as stereotactic magnetic resonance-guided adaptive radiotherapy (SMART). SMART is an advanced SBRT modality that is currently being utilized for many tumor types in clinics around the globe to improve therapeutic efficacy and safety [17,19]. The global adoption of this novel MRL technology for SMART continues to accelerate. This has led to a multitude of innovative trials and registries [19,20] that explore and expand the impact of this new treatment modality. Table 1 lists all currently active trials exploring either nonadaptive MRL-based SBRT (MRL-SBRT) and SMART registered on ClinicalTrials.gov, accessed on 12 March 2023.

**Figure 2:**
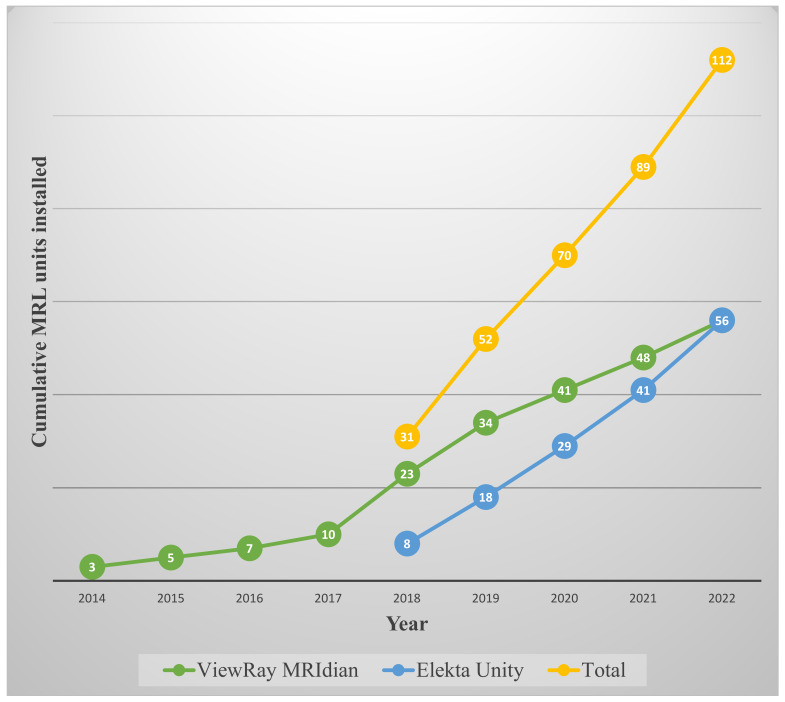
Cumulative installations of ViewRay MRIdian and Elekta Unity MRLs over time. ViewRay MRIdian was initially a tri-^60^Co system, with MRL installations beginning in 2017. All existing ViewRay MRIdian systems, except for one, have been upgraded to MRLs. Elekta Unity systems were initially pre-clinical until late 2019. All existing Elekta Unity systems have been upgraded to fully clinical systems. Data used for the creation of Figure 2 were directly provided by Elekta and ViewRay team members.

**Figure 3:**
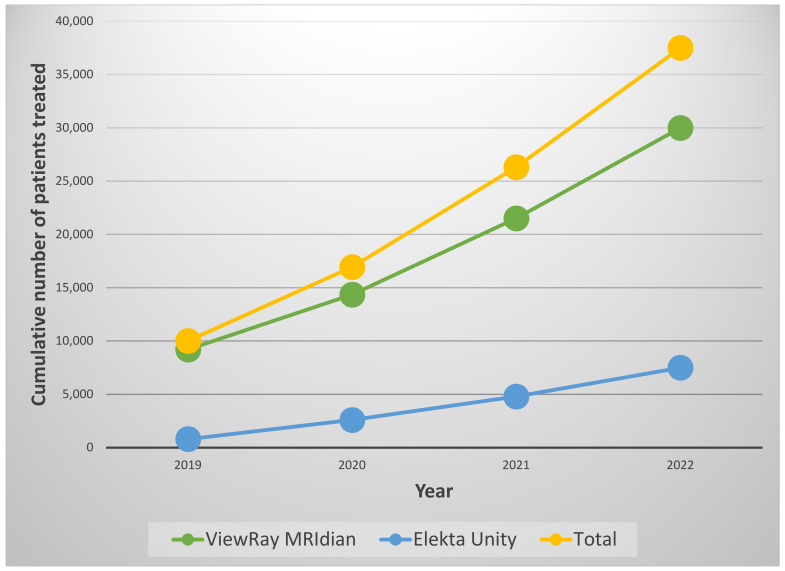
Cumulative treatments of ViewRay MRIdian and Elekta Unity MRLs per year since 2019. Data used for the creation of Figure 3 were directly provided by ViewRay and from data presented at the 9th annual MR in RT symposium [25].

The two most common commercially available MRLs are the ViewRay MRIdian (ViewRay Technologies Inc., Oakwood Village, OH, USA) and Elekta Unity (Elekta AB, Stockholm, Sweden). The global adoption of MRL technology has been driven by these 2 systems, with 112 (56 of each) ViewRay MRIdian and Elekta Unity systems having been installed as of 31 December 2022 (Figure 2). Since 2019, these systems have combined to perform an estimated 37,500 treatments (Figure 3). The MRIdian system combines a 0.345 T-field strength split-bore magnet MRI with a 28 cm gap that contains the 6 MV flattening filter-free (FFF) linear accelerator components [21]. ViewRay originally produced a tri-^60^Co unit; however, these have all been upgraded (except for one) to MRL [22]. The Elekta Unity combines a 1.5 T MRI (Philips, Amsterdam, The Netherlands) and a 7 MV FFF linear accelerator irradiating through a cryostat [16]. Although both ViewRay MRIdian and Elekta Unity are MRLs and can be utilized for the purposes of SMART, there are important distinctions between the two machines regarding their capabilities. The most obvious difference is the conventional (i.e., 1.5 T) static magnetic field (B_0_) strength of Elekta Unity, as compared to the low-field (i.e., 0.345 T) MRIdian system. Higher B_0_ improves the signal-to-noise ratio and generally improves overall image quality. However, the relationship between the field strength and contrast-to-noise ratio, which is important for target-tracking, is not straightforward [23]. The higher B_0_ also makes multiparametric imaging easier to perform as well as provides the general capability to immediately utilize pulse sequences developed for diagnostic MRI purposes at the same field strength. However, since both system-specific and patient-induced (e.g., chemical shift and magnetic susceptibility effects) geometric distortion also scales with B_0_, it is easier to manage in the low-field machine [13,24]. Lastly, the MRIdian system has had real-time tumor-tracking with automatic beam-gating since its launch, whereas the Unity system achieved FDA approval for tracking on 28 February 2023.

Despite the improvements in personalized radiotherapy already achieved by MRLs, their full potential is not yet realized. MRLs could enable significant strides in personalized cancer therapy by analyzing the daily MR images for subtle intra- and peri-tumor anatomical and physiological/functional changes in response to ablative doses. The ability to identify and determine the clinical significance of the tumoral response during each fraction could be exploited for further individualized plan adaptation [26,27,28]; Therefore, these MRI radiomic features could allow for online biological and physiological, in addition to the current morphological, online plan adaptation in the future.

In this review, we summarized current and potential future directions for SMART clinical applications and trials, by cancer type. Although we only focused on sites that could benefit the most from SMART, this review was not comprehensive in scope. We focused on as many sites as possible where SMART has been actively improving care and has evidence of improvement over CT-based SBRT. In addition, in a separate section, we explored how existing technologies could potentially be integrated with current MRL systems to significantly improve personalized radiotherapy.

## 2. SMART Clinical Applications

### 2.1. Head and Neck Cancer

MRI plays an important role in the diagnosis and treatment of head and neck cancers (HNCs) due to the improved visualization of the muscle invasion, the perineural invasion, and the extracapsular extension [29,30]. Therefore, MRI could improve target delineation and expand the role of adaptive RT in these cancers [31]. Early data has suggested that an offline adaptation with MRL could be efficacious [32]. The limited evidence on the treatment of HNC utilizing the tri-^60^Co system demonstrated effective tumor control with low toxicity rates [33,34]. The early evidence on the treatment of HNC using an MRL demonstrated similar feasibility and safety [19,35,36]. An early report from the MOMENTUM study (NCT04075305) demonstrated the feasibility of MRgRT with a 1.5 T MRL in 13 patients with HNC [19]. These initial data have helped establish the feasibility of conventionally fractionated HNC radiotherapy using MRLs. SBRT has become an important tool for radiation oncologists in the treatment of many types of de novo and recurrent HNCs, although concerns remain regarding toxicity and appropriate tumor selection [37,38]. The advantages of SMART over conventional SBRT modalities could expand the therapeutic window of HNC SBRT. Currently, there is a prospective early-phase trial exploring SMART feasibility and safety for HNC utilizing the 1.5 T MRL (NCT04809792) that is expected to complete enrollment in late 2023.

### 2.2. Central and Ultra-Central Lung Tumors

SBRT is part of the standard of care for early-stage, non-operable non-small-cell lung cancer (NSCLC) [39] and has been commonly used to treat metastatic lesions in the lungs [40,41]. Lung SBRT has been demonstrated to have excellent local control and minimal toxicity rates [42,43,44]. However, concerns remain for using SBRT on more centrally located lung lesions due to high rates of toxicity [45]. These central lesions, defined as being within two cm of the proximal bronchial tree (PBT) by the Radiation Therapy Oncology Group (RTOG) [46], and ultra-central (UC) lesions, defined as being within one cm of the PBT, have had significantly higher rates of SBRT-related grade-3–5 toxicity, as compared to more peripherally located tumors [45,46,47,48,49]. Up to one-third of patients with UC lung tumors have experienced grade-3 or higher SBRT-related toxicity, and 15% died as a result of the treatment [49]. These high rates of toxicity were likely related to the uncertainty of the large internal target volume (ITV) and soft-tissue organs at risk (OARs) in the positional planning with CT-based SBRT, leading to unintentionally high doses delivered to the PBT.

SMART has overcome these limitations with the use of MR-guided online plan adaptation to push unacceptably high doses away from OARs and real-time tumor-tracking to control for respiratory motion during treatment [50,51,52,53,54,55]. SMART for central and UC lesions has been associated with local control rates approaching 96% for both primary and metastatic cancers [53]. In addition, the toxicity rates were comparable to those in peripheral lesions [53,54]. Importantly, recent evidence did not correlate the risk of late intrapulmonary hemorrhage with SMART [56], which was a primary cause of treatment-related death [49] with CT-based SBRT. These initial experiences led to the development of multiple prospective studies exploring SMART for central and ultra-central lesions. Trials such as LUNG Stereotactic Adaptive Ablative Radiotherapy (LUNG STAAR; NCT04917224) Stereotactic Radiotherapy for Centrally Located Lung Tumors (STRICT-LUNG STUDY; NCT04917224); and Ultra-Centrally Located Lung Tumors (STAR-LUNG STUDY; NCT05354596) are exploring the clinical outcomes of SMART for primary early-stage NSCLC and metastatic lesions.

### 2.3. Cardiac Metastases

The heart and pericardial tissues are rare sites of malignancy, with the most generous estimates of the incidence of primary and metastatic lesions being ≤0.03% and ≤3%, respectively [57,58]. As survival continues to improve in the metastatic setting, particularly in melanoma, the incidence of cardiac metastases has increased [59,60]. The surgical resection of these lesions has traditionally been the only means of definitive therapy [61], with RT playing a purely palliative role [62]. Advances in the field of radiation oncology have indicated the feasible effective treatment of these lesions with SBRT [63]. SMART has the potential to improve the delivery of SBRT to these highly mobile lesions that have been difficult to identify with CT imaging. Currently, SMART data are very limited for these rare tumors. A single institutional experiment in five patients with cardiac lesions that were treated with SMART reported excellent tumor coverage and minimal toxicity [64]. Larger series are required to optimize the dosage for various histologies and to better explore the role of MRgRT in cardiac tumors.

### 2.4. Pancreatic Cancer

The role of SBRT in borderline resectable (BRPC) and locally advanced pancreatic cancer (LAPC) has been controversial [65,66,67,68,69,70]. Although SBRT appeared to significantly improve local control, the concerns regarding the lack of improvement in overall survival and toxicity have persisted [65,66,67,68,69,70,71,72]. Data suggested that the dose escalation could have been associated with the improvements in both local control and overall survival [73,74,75,76,77,78]. Dose-escalated SBRT has historically been limited in practice due to the radiosensitive gastrointestinal organs that surround the pancreas. However, SBRT via SMART could overcome these toxicity-related challenges in pancreatic cancer due to the excellent soft-tissue visualization and online plan adaptation and gating [79,80,81].

Ablative SMART (A-SMART) demonstrated an excellent safety profile [82,83] and even appeared to be an effective option for elderly patients with unresectable pancreatic cancer who were at increased risk for treatment-related toxicities [84]. Initial studies exploring A-SMART for BRPC and LAPC demonstrated limited toxicity and improved clinical outcomes, with local control and overall survival rates approaching 90% and 70%, respectively [80,81,83,85,86,87]. In addition, pre-operative A-SMART for BRPC patients was associated with excellent negative resection rates and did not appear to increase the intra- or post-operative mortality [88]. The results of the multicenter phase-II trial, SMART for Locally Advanced Pancreatic Cancer (NCT03621644), were recently presented and demonstrated a median overall survival of 22.5 months and a 1-year overall survival of 94% [89]. The incidence of grade-3 or higher toxicity related to A-SMART was 2.2%. Due to these positive results, a phase-III trial has been announced, the Locally Advanced Pancreatic Cancer Treated with Ablative Stereotactic MRI-guided Adaptive Radiation Therapy (LAP-ABLATE) trial (NCT05585554), which will compare the standard chemotherapy to sequential chemotherapy, followed by A-SMART. Additional phase-II clinical trials exploring SMART for pancreatic pain control in metastatic disease (NCT05114213), SMART in frail and elderly patients (NCT05265663), a combination of intensified sequential chemotherapy with A-SMART (NCT04570943), and SMART for neuroendocrine pancreatic tumors (NCT05037461) are ongoing.

### 2.5. Liver Tumors

Surgical resection is the standard of care for primary hepatocellular carcinoma (HCC) [90] and hepatic oligometastases [91,92]; however, only one-fifth of patients are deemed eligible for surgery [93]. For unresectable hepatic tumors, SBRT could be a potential treatment option that has the advantage of not being an invasive procedure [94,95,96]. Over three years, SBRT achieved local control rates of over 90% for metastatic lesions, if treated with ablative doses [97]. Due to the parallel architecture of the liver, it can withstand high doses of radiation in small areas but is at high risk of radiation-induced liver disease (RILD) with larger targets [98]. In addition, the local radiosensitive gastrointestinal organs are at high risk of toxicity during liver irradiation. SBRT, in particular, has been associated with a risk of grade-3 or higher toxicity in up to one-third of patients [99], thus limiting patient selection and dose escalation. However, MR-guided SBRT can overcome many of the challenges faced by CT-based SBRT.

SMART has reduced irradiated liver volumes without an ITV (on some MRL systems that provide patient anatomy tracking/gating) and tighter PTV margins and ensured tolerances for nearby radiosensitive structures were safely and reliably respected while achieving the requisite ablative doses [50,87,100,101,102]. Patients have also forgone the need for invasive fiducial markers for gating and tracking with SMART. SMART for primary and metastatic liver lesions has been demonstrated to have local control rates between 75% and 100% at 21 months with a grade-3 toxicity rate of only 8% and no grade-4 toxicity or treatment-related deaths [101]. These initial reports of SMART in hepatic lesions are promising but limited due to their retrospective nature and short follow-up periods. Multiple trials exploring liver-focused SMART have been initiated to better define its role. The phase-II Magnetic Resonance-Guided Adaptive Stereotactic Body Radiotherapy for Hepatic Metastases (MAESTRO) randomized trial is currently recruiting patients to compare ITV-based SBRT and SMART. The Adaptative MR-Guided Stereotactic Body Radiotherapy of Liver Tumors (RASTAF) phase-II trial (NCT04242342) is exploring dose escalation of up to 60 Gy in 5 fractions with SMART in all types of liver tumors. The OAR-Based, Dose-Escalated SBRT With Real-time Adaptive MRI Guidance for Liver Metastases trial (NCT04020276) is a 2-staged phase-I study that is exploring dose escalation of up to 80 Gy in a 4-plus-4 with a confirmatory expansion cohort design.

### 2.6. Adrenal Metastases

The adrenal gland is a common site of metastases from many malignancies [103] and the indications have been increasing for a definitive treatment in metastatic adrenal lesions [104,105]. There was insufficient evidence to determine the best local treatment modality for isolated and limited adrenal metastases [106]. While surgery is a curative modality option for isolated adrenal metastasis, it has often been contraindicated in the presence of more extensive disease, in elderly patients, and in those with other significant co-morbidities [103,107,108]. Additionally, the recovery time of these procedures usually requires lengthy hospital stays [103]. SBRT is a valid alternative when surgery is not feasible [106,109,110,111]. However, patients have historically presented significantly worse tumor control, as compared to adrenalectomy [108]. This has likely been due to dose limitations in conventional CT-based SBRT because of the interfractional movement of OARs [112,113], which can be up to 3 cm for local radiosensitive gastrointestinal organs, as well as intrafractional respiratory-induced movement [114]. However, a BED_10_ of >100 Gy was associated with tumor control approaching that of a resection [111,115]. SMART was capable of respiratory-motion management and online plan adaptation for positional changes in local OARs, making it feasible for the delivery of ablative doses. The early data supported the feasibility and the efficacy of SMART in these tumors [86,116]. The recent data has supported this approach by demonstrating 1-year local control rates of 100% in a limited series [117]. The MRL Dana–Farber master trial (NCT04115254) and the SMART-ONE trial (NCT03878485) will help define the feasibility and the role of SMART for adrenal SBRT.

### 2.7. Kidney Cancer

The role of radiotherapy and SBRT has been limited in the treatment of primary kidney cancer [118]. SBRT could offer a benefit in large tumors (>4 cm) that are not suitable for surgical resection [119]. SBRT appeared to demonstrate exponential cell death in renal cell carcinoma, as compared to conventional fractionation [120]. However, CT-based SBRT often must use large margins [121] to account for movements during therapy [122]. MRL-based SBRT had an advantage over CT-based SBRT by eliminating the need for ITVs, one of the reasons for large margins [123]. The early data has suggested that SMART could be well tolerated with clinically meaningful disease control [124,125]. Therefore, if currently active trials establish a larger role of SBRT [126,127], SMART could play an important part in kidney cancer radiotherapy in the future.

### 2.8. Breast Cancer

Breast conservation is important to many people with breast cancer, and treatment strategies to avoid mastectomies have been developed that are effective and widely adopted for early-stage breast cancer. RT played an integral role in this treatment design to ensure the clinical outcomes were similar to that of mastectomy [128]. Due to concerns of normal tissue exposure and the inconvenience of 5–6 weeks of daily RT in traditional post-partial mastectomy whole-breast RT, accelerated partial-breast irradiation (APBI) was explored as a potential alternative in specially selected patients with favorable patient and tumor characteristics [129,130]. APBI focuses solely on the areas surrounding the surgical bed and is typically delivered within 1–2 weeks. Both brachytherapy and external beam techniques were explored to determine their unique advantages and drawbacks [131,132]. Brachytherapy offers excellent conformality but is a more invasive procedure. External beam radiotherapy (EBRT) is non-invasive but requires larger margins due to the uncertainties in the daily design and the intrafractional motion management. The high dose per fraction for EBRT ABPI could have contributed to late cosmetic toxicity, although evidence for this has been mixed [131,133,134], with a larger percentage of treated breast volume being a predictor for adverse cosmetic outcomes [135]. SMART could be an excellent external beam APBI modality to improve clinical outcomes. SMART could improve upon existing external beam ABPI due to its superior soft-tissue visualization of the resected cavity and online plan adaptation for the daily design that could allow for a smaller PTV, or even a zero-margin PTV, without sacrificing coverage.

A single institution prospective trial of a 10-fraction with zero-margin PTV APBI on a 0.35 T MRL in 30 patients reduced treatment volumes by 52%, as compared to conventional APBI [136]. These data supported the exploration of APBI delivered with SMART. An early dosimetric analysis demonstrated that 88.5% of the possible dosimetric objectives were fulfilled during planning [137]. The early evidence of APBI delivered with MRLs demonstrated dosimetric advantages over traditional CT-based strategies. If long-term clinical and cosmetic outcome data for APBI delivered with SMART are favorable, this could become an important modality for elderly people with early-stage breast cancer, as approximately 40% of these patients are unable to complete their 5-year hormone therapy, which significantly increases the risk of disease recurrence [138]. However, the clinical benefit of SMART APBI remains unclear, as long-term outcomes for CT-based APBI are excellent. Therefore, whether the dosimetric advantages translate into clinically meaningful improvements over existing APBI techniques is not yet known. The phase-II trial, Real-Time MRI-Guided Three-Fraction Accelerated Partial-Breast Irradiation in Early Breast Cancer (MAPBI) (NCT03936478), is exploring cosmetic and clinical outcomes with SMART APBI.

### 2.9. Prostate Cancer

There has been an increased utilization of SBRT to reduce the length of treatment and take advantage of the low α/β ratio in prostate cancer [139,140]. The early studies demonstrated significant gastrointestinal and genitourinary toxicity [141,142]. Recent large phase-III trials have had conflicting evidence regarding toxicity [143,144]. MR-guided SBRT (Non-MRL based) is one strategy that has been employed to reduce toxicity. MRI is regularly used in the diagnosis, staging, and management of prostate cancer [145,146] due to its excellent visualization of lesions in both the prostate and the normal surrounding tissue [147]. MRI has been used during treatment planning to better visualize the critical OARs [148], to aid in contouring, and more recently, to help guide boosters to high-risk foci [149]. Therefore, nonadaptive MRL-SBRT and SMART appear to be a logical evolution in prostate SBRT [150,151].

SMART and nonadaptive MRL-SBRT offer multiple advantages over CT-based prostate SBRT, which includes include inter- and intra-fractional rectal motion management and proper daily alignment for urethral-sparing techniques. In addition, SMART and nonadaptive MRL-SBRT do not require the invasive implantation of fiducial markers for daily alignment, which is often a transrectal procedure that has been associated with complications that impacted the quality of life in up to one-third of patients [152,153]. SMART feasibility for prostate cancer is well established [154,155,156]. Urethral-sparing techniques demonstrated significantly low rates of acute genitourinary toxicity [157]. The results from the SCIMITAR trial, a phase-II, dual-center, single-arm trial that treated post-operative prostate cancer at high-risk for recurrence, with SBRT, demonstrated worse gastrointestinal toxicity of up to 6 months in patients treated with CT-based SBRT, as compared to MRL-SBRT [158].

The MIRAGE trial (NCT04384770) was the first phase-III trial to compare SMART with CT-based SBRT [159]. MIRAGE sought to evaluate if the aggressive margin reduction that had been made feasible with MRL-based treatment would significantly reduce acute grade-2 or higher genitourinary toxicity, as compared to CT-guided treatment [159]. MRL-based MRgSBRT demonstrated a significant reduction in grade-2 or higher acute genitourinary (24.4% (95% CI, 15.4–35.4%) vs. 43.4% (95% CI, 32.1–55.3%); *p* = 0.01) and gastrointestinal (0.0% (95% CI, 0–4.6%) vs. 10.5% (95% CI, 4.7–19.7%); *p* = 0.003) toxicity [159]. This first prospective head-to-head study of CT-based SBRT and MRL-based MRgSBRT clearly demonstrated how MRL capabilities could translate into improved clinical outcomes.

There are multiple current phase-II trials exploring SMART in prostate cancer. The European Stereotactic MRI-Guided Radiation Therapy for Localized Prostate Cancer (SMILE) trial (NCT04845503) is exploring SMART feasibility within an estimated cohort of 68 males. In addition, a phase-II trial (NCT05183074) is exploring the utilization of an MRL to deliver SMART with simultaneously integrated boosters for MR-prominent tumor foci. Another phase-II trial (NCT04984343) is exploring SMART hypofractionation, reducing the standard 5 fractions to 2, to continue reducing treatment time in this very common cancer.

### 2.10. Spinal Metastases

Spine RT is an important part of metastatic disease management to improve pain, prevent pathological fractures, and prevent neurological morbidity. SBRT had improved efficacy, as compared to conventional forms of radiotherapy [160]. MRIs have been used in spine SBRT to accurately delineate the spinal cord and create a 1–2 mm planning OAR volume (PRV) to decrease disease coverage [160]. Bony structures act as surrogates for the daily design in conventional CBCT image guidance, but CBCTs are not reliable for the accurate visualization of the spinal cord. Therefore, a spinal cord PRV is created during treatment planning to account for daily motion management. MRLs could provide a benefit due to their superior demarcation of the spinal cord and other soft-tissue OAR positions with daily MRIs, as compared to CBCTs [10]. Dosimetric feasibility studies suggested that design improvements with MRI could reduce the dose to the spinal cord [161]. Daily MRIs allow for direct plan registration of the spinal cord, thereby eliminating the need for cord PRVs and allowing for greater tumor coverage. Additionally, the comparatively low fields of the MRLs, as compared to many diagnostic MRIs, have also decreased the artifact and geometric distortions caused by metal hardware [162]. Utilizing an MRL for spine SBRT also improved the integration of the CT treatment planning scan because the radiation oncologist was able to ensure the same patient position [163,164]. These advantages could allow for reduced margins and safe dose escalation. However, it remains unclear if these dosimetric advantages will be clinically meaningful, as compared to CBCT-based spine SBRT. The results of a current phase-I/II trial treating all sites of disease with SMART, including the spine (NCT04115254), and the Pilot Study of Same-Session MR-Only Simulation and Treatment with SMART for Oligometastases of the Spine (NCT03878485) could help determine the feasibility of this technique.

### 2.11. Oligometastatic Cancer

The increasing data have demonstrated that patients with limited metastases who were treated in a definitive manner at all sites of disease had increased overall survival [165]. This limited metastatic state is termed oligometastatic, and it blurs the line between localized and incurable systemic disease. Recent clinical trials have demonstrated the benefit of SBRT for patients with oligometastatic cancer, typically defined as between one and five metastatic lesions. Randomized phase-II studies of oligometastatic NSCLC [166] and prostate cancer [167] showed improved outcomes with SBRT at all metastatic sites. The phase-II SABR-COMET trial demonstrated that SBRT had improved overall and progression-free survival for various histologies, as compared to standard palliative therapy [168]. However, multi-site SBRT has a significant risk of increased toxicity. The NRG BR-001 trial that delivered SBRT to all sites of metastatic disease demonstrated a rate of late grade-3 or higher toxicity to be 20% at 2 years [169]. Similarly, SABR-COMET reported a 29% rate of grade-2 or higher toxicity, including 3 treatment-related deaths, in the SBRT group, as compared to only 9% in the control group. SMART was uniquely suited for delivering high-dose SBRT to multiple sites concurrently due to its excellent therapeutic window [170]. In addition, SMART has also enabled safe isotoxic dose escalation [82,86], with increased local control and overall survival rates.

Data have been limited concerning the use of SMART in an oligometastatic setting, but SMART has been well tolerated [86,102,171]. Several ongoing clinical trials are evaluating the use of MRgRT in the management of oligometastatic disease. Notably, the SMART-ONE trial is a single-arm trial that is investigating the feasibility of delivering single-fraction MR-guided SBRT to up to 10 sites of disease (NCT04939246). The Washington University School of Medicine is exploring the use of SMART in oligometastatic disease of the spine (NCT03878485). We eagerly await the results of these trials to establish the feasibility, efficacy, and safety of MRgRT in an oligometastatic setting.

### 2.12. Ablative Dose Re-Irradiation

Re-irradiation (reRT) has historically been limited due to the increased risk of severe toxicity due to cumulatively high OAR doses; however, it also could provide a significant benefit in carefully selected patients with locally recurrent or progressive cancer [172,173]. Therefore, dose selection in reRT is a delicate balance between prioritizing tumor control and patient safety that usually results in modest dose delivery. Historically, these doses did not offer robust local control, especially in patients who did not proceed to surgery [174]. However, dose escalation could improve long-term local control and overall survival in reRT [174,175,176]. The improved therapeutic ratio of SMART could allow for the safe delivery of dose-escalated reRT.

SMART reRT data have been limited, but the treatment has been well tolerated. SMART reRT in the abdomen and pelvis demonstrated a 1-year local control rate approaching 90% [177]. With a median follow-up of 14 months, there was no acute or late grade-3 or higher toxicity, demonstrating the safety of this modality. Another recent report focused only on prostate reRT and demonstrated a 1-year disease progression-free survival rate that also approached 90%, while maintaining minimal toxicity [178]. SMART reRT appears to be associated with strong local disease control and minimal toxicity, which could warrant further investigation in clinical trials.

## 3. Future Directions

SMART has enabled the delivery of greater doses to tumors surrounded by some of the most radiosensitive normal tissue within the body, and this has indicated potential dose-escalated treatments that were previously thought to be infeasible, as discussed. Although this has been primarily achieved with MR-guided anatomic adaptation, we believe that the future of SMART may lie in advanced adaptation techniques. This requires the immense data stored in daily MRIs to better understand the tumoral response to treatment throughout the course of radiotherapy, and then these daily insights must be used to adjust both the dosage and fractions throughout the treatment. This would represent major a paradigm shift in the field of radiation oncology. Traditionally, dosage and fractionations were determined prior to and during treatment planning. Even with the current advances in SMART, we continue to use this approach and merely adapt to improve the delivery of a predetermined dose and fractionation. However, studying the tumor changes in response to treatment via daily MRI could provide deeper insights into the nature of a specific tumor and how it will ultimately respond to the current dose and fractionation plan.

Two novel studies, Adaptive Radiation for Locally Advanced Rectal Adenocarcinoma (NCT05108428) and Theragnostic Utilities for Neoplastic Diseases of the Rectum by MRI-Guided Radiotherapy (THUNDER2; NCT04815694), are already utilizing MRL to explore plan adaptation based on tumoral response. They are relying on the tumoral volumetric changes to identify which rectal tumors would benefit the most from sequential booster-dose escalation. Guiding treatment planning based upon volumetric response for certain cancers is clinically feasible when using MRL in longer treatment courses of conventional and minimally hypofractionated radiotherapy. However, utilizing this same technique with SMART is far more difficult due to the significantly shorter treatment course that often does not allow enough time for tumors to demonstrate a clinically obvious volumetric response. Therefore, the more subtle and less well-understood peri- and intra-tumoral changes during therapy should be utilized to guide physiologically and biologically adaptive radiotherapy. The multiparametric MRI (mpMRI) allows for a wider breadth of imaging data to better investigate these often-imperceptible changes.

### MRL-Based Multiparametric MRI

MRL adaptation has traditionally been employed for the management of interfractional tumoral and OAR changes in shape and position. However, MRI has also been used for assessing biological and physiological information [179,180,181], as well as for MRI techniques termed mpMRI [182]. One such technique is diffusion-weighted imaging [183] which enabled the detection of changes in water mobility [184]. These changes were correlated with tumor growth [185] and necrosis [186]. This was facilitated by mapping a parameter known as the apparent diffusion coefficient (ADC), which was then used to track the response to radiation therapy [187]. ADC mapping is particularly attractive in adaptive radiotherapy since the changes in ADC could be noted before morphological changes in the tumor [188], and these changes in diffusion could be used to guide dose-escalation strategies and biologically guided radiation plan adaptation [189,190]. Diffusion-weighted imaging has been applied using a 1.5 T linear accelerator [191,192,193]. Although technical challenges have been reported [194], DWI was included as an option in this simulation [35]. DWI was initially applied using the 0.35 T tri-^60^Co system [195,196] and was shown to be predictive of tumor histology [197] and, in combination with deep learning, therapeutic response [198]. Technical challenges were reported [199] when the 0.35 T MRgRT system had transitioned from the tri-^60^Co system to MRL, but recent applications using DWI with a 0.35 T MRL have appeared promising [200].

A potential application of MRL-based adaptive radiotherapy is the use of metabolic changes to guide RT, as this has been utilized in the recently developed PET/CT-guided radiotherapy delivery systems [201]. Cancer metabolism is severely dysregulated [202], and this dysregulation is reflected downstream, as the concentrations of many metabolites are modulated in cancer cells [203]. While positron-emission tomography (PET) [204,205,206] has traditionally been applied to observe the metabolic accumulation in tumor cells, MR-based techniques, such as magnetic resonance spectroscopic imaging (MRSI) [207], chemical exchange saturation transfer (CEST) [208,209], and hyperpolarized dynamic magnetic resonance spectroscopy [210], were able to interrogate metabolic processes further downstream [211]. MRSI allowed for the noninvasive mapping of a number of metabolite concentrations by simultaneously acquiring MR data in the spatial frequency and temporal domains [212]. It was applied to produce high-resolution metabolite maps in gliomas [213], and lactate mapping of glioblastoma has been performed using deuterium [214]. Additionally, using phosphorus-based MRSI, the mapping of intra- and extra-cellular pH in tumors was demonstrated [215]. The technical limitations concerning the online incorporation of MRSI with MRL as a work flow have persisted due to the relatively long scan times [216] and low sensitivity in conventional magnetic-field-strength systems [217]. Sensitivity could be counteracted by hyperpolarizing the nucleus, which has resulted in a large increase in sensitivity for a short period of time [218]. The main application has been to observe the dynamic conversion of pyruvate into lactate in tumors [219]. Lastly, CEST allowed for the indirect detection of low concentration solutes via their effect on the water MR signal [220]. CEST has been shown to predict the chemo-radiotherapeutic response of tumors [221,222].

While these MR-based metabolic-imaging techniques have yet to be incorporated into online MRgRT due to the technical challenges, they have significant potential for assessing biological behavior in adaptive RT. The additional incorporation of artificial intelligence into the interpretation of mpMRI data could facilitate biologically driven RT plan adaptation [223,224].

## 4. Barriers and Limitations

Although MRLs represent one of the most exciting advancements within the field of radiation oncology, these combined linear accelerators have limitations. This novel technology is resource intensive, requiring considerable financial and time investments for operation. The commissioning of MRL requires the development of departmental MR-safety protocols similar to those for diagnostic MRIs, which include MRI safety questionnaires for all patients and thorough MRI safety training for all users with an emphasis on ferrous-material awareness [225]. MRL uses a different workflow, as compared to other linear accelerators; thus, all members of the treatment team, including the physician, physicists, and therapists, must learn to properly operate MRLs [226]. Furthermore, the daily time requirement for online adaptive radiotherapy can be substantial, from 30 to 60 min per treatment, to allow for adequate plan evaluation, adaptation, and treatment delivery, even with an experienced team. This limits total patient throughput and can often require considerable time-at-machine for physicians and physicists.

MRLs also have physical limitations due to the special physics of concurrent MRI with external beam radiotherapy. Lorentz forces have resulted in overdosing hollow organs and required an advanced treatment planning system [227]. MRI geometric distortion, the uncertainty associated with MRI regarding radiation isocenter distance, the multi-leaf collimator position error, and the uncertainties in voxel size and tracking have presented additional physical limitations [101]. Therefore, the familiarity and expertise of physicians, dosimetrists, and physicists regarding these special physics were required for optimal treatment planning and establishing more robust quality assurance methods [86]. MRIs lack electron density and attenuation coefficient information. Therefore, CT images are still required for treatment planning. Additionally, there is a lack of a six-degree couch for adjustments due to the confined space of the MRLinac system.

Patient selection is critical for a successful MRL program. Special attention is required for patients with claustrophobia, large body habitus, and MRI-incompatible implanted devices. Patients with claustrophobia may require pre-treatment anxiolytic therapy or may not be able to tolerate it at all. Patients with large body habitus may not be able to fit within the geometric dimensions of the machine. Even if the patient physically fits into the machine, they may exceed the maximal field-of-view, which can result in aliasing artifacts. This is especially important when using special devices, such as coils, depending on the treatment site.

Diligent screening for all potentially implanted ferromagnetic devices is required for all patients, and alternative treatment options should be considered in these cases.

MRL has many advantages over CT-based linear accelerators. However, MRL was not designed to be a replacement for CT-based linear accelerators. We found that MRL was best suited in cases where its unique advantages were required to deliver a treatment that would be too dangerous in a CT-based linear accelerator.

## 5. Conclusions

MRL is rapidly becoming an integral instrument for personalized radiotherapy. SMART represents the next generation of SBRT by expanding the therapeutic window due to its vastly improved precision through enhanced soft-tissue resolution and daily MR-guided online adaptation, along with real-time gating in MRIdian. Safe dose escalation using isotoxic approaches with SMART appears to be improving disease outcomes across multiple tumor sites. There are a multitude of cutting-edge clinical trials currently in progress to establish this new modality’s role in many types of cancer. Looking forward, MRL and mpMRI appear to have significant synergistic potential, in conjunction with SMART, in personalized cancer therapy.

## Figures and Tables

**Table 1:** Active SMART and nonadaptive MRL-SBRT clinical trials registered on ClinicTrials.gov. Both actively recruiting and active but not-yet-recruiting trials were included.

Study Title	Sponsor	Site	Condition/Disease	Estimated Enrollment	ClinicalTrials.gov Identifier
A Master Protocol of Stereotactic Magnetic Resonance Guided Adaptive Radiation Therapy (SMART)	Dana–Farber Cancer Institute	All/Multiple sites	N/A	1000	NCT04115254
The MR-Linac Technical Feasibility Protocol (UMBRELLA-II)	The Netherlands Cancer Institute	All/Multiple sites	N/A	140	NCT04351204
The Multiple Outcome Evaluation of Radiation Therapy Using the MR-Linac Study (MOMENTUM)	UMC Utrecht	All/Multiple sites	N/A	6000	NCT04075305
Magnetic Resonance Guided Radiation Therapy (CONFIRM)	Dana–Farber Cancer Institute	All/Multiple sites	Gastric Cancer, Invasive Breast Cancer, in Situ Breast Cancer, Mantle Cell Lymphoma, Larynx Cancer, Bladder Cancer	70	NCT04368702
Immune Checkpoint Inhibitor and MR-guided SBRT for Limited Progressive Metastatic Carcinoma	Baptist Health South Florida	All/Multiple sites	Metastatic tumors	52	NCT04376502
Stereotactic MRI-guided Adaptive Radiation Therapy (SMART) in One Fraction (SMART-ONE)	Baptist Health South Florida	All/Multiple sites	Oligometastatic cancer, up to 10 sites of disease	30	NCT04939246
Real-Time MRI-Guided 3-Fraction Accelerated Partial Breast Irradiation in Early Breast Cancer (MAPBI)	University of Wisconsin, Madison	Breast	Breast Cancer, DCIS	30	NCT03936478
MR-Linac Guided Adaptive FSRT for Brain Metastases From Non-small Cell Lung Cancer	Sun Yat-Sen University	Central Nervous System	Brain Metastases from Non-Small Cell Lung Cancer	55	NCT04946019
Pilot Study of Same-session MR-only Simulation and Treatment With Stereotactic MRI-guided Adaptive Radiotherapy (SMART) for Oligometastases of the Spine	Washington University School of Medicine	Central Nervous System	Oligometastases of the Spine	10	NCT03878485
Locally Advanced Pancreatic Cancer Treated With ABLAtivE Stereotactic MRI-guided Adaptive Radiation Therapy (LAP-ABLATE)	ViewRay Inc.	Gastrointestinal	Pancreatic Cancer	267	NCT05585554
Sequential Treatment With GEMBRAX and Then FOLFIRINOX Followed by Stereotactic MRI-guided Radiotherapy in Patients With Locally Advanced Pancreatic Cancer (GABRINOX-ART)	Institut du Cancer de Montpellier—Val d’Aurelle	Gastrointestinal	Pancreatic Cancer	103	NCT04570943
MR-Guided Adaptive SBRT of Primary Tumor for Pain Control in Metastatic PDAC (MASPAC)	Ludwig-Maximilians—University of Munich	Gastrointestinal	Pancreatic Cancer	92	NCT05114213
Stereotactic Radiotherapy vs. Best Supportive Care in Unfit Pancreatic Cancer Patients (PANCOSAR)	Amsterdam UMC	Gastrointestinal	Pancreatic Cancer	98	NCT05265663
Precision Radiotherapy Using MR-linac for Pancreatic Neuroendocrine Tumours in MEN1 Patients (PRIME)	J.M. de Laat	Gastrointestinal	Pancreatic Neuroendocrine Tumors	20	NCT05037461
MR-guided Pre-operative RT in Gastric Cancer	Washington University School of Medicine	Gastrointestinal	Gastric cancer	36	NCT04162665
Magnetic Resonance-guided Adaptive Stereotactic Body Radiotherapy for Hepatic Metastases (MAESTRO)	University Hospital Heidelberg	Gastrointestinal	Liver Metastases	90	NCT05027711
OAR-Based, Dose Escalated SBRT With Real-time Adaptive MRI Guidance for Liver Metastases	University of Wisconsin, Madison	Gastrointestinal	Liver Metastases	48	NCT04020276
Adaptative MR-Guided Stereotactic Body Radiotherapy of Liver Tumors (RASTAF)	Centre Georges Francois Leclerc	Gastrointestinal	Liver Metastases	46	NCT04242342
Radiotherapy With Iron Oxide Nanoparticles (SPION) on MR-Linac for Primary & Metastatic Hepatic Cancers	Allegheny Singer Research Institute	Gastrointestinal	Liver tumors	25	NCT04682847
Stereotactic MRI-guided Radiation Therapy for Localized Prostate Cancer (SMILE)	University Hospital Heidelberg	Genitourinary	Prostate Cancer	68	NCT04845503
Randomized Trial of Five or Two MRI-Guided Adaptive Radiotherapy Treatments for Prostate Cancer (FORT)	Weill Medical College of Cornell University	Genitourinary	Prostate Cancer	136	NCT04984343
MR-linac Guided Ultra-hypofractionated RT for Prostate Cancer	Chinese Academy of Medical Sciences	Genitourinary	Prostate Cancer	50	NCT05183074
Randomized Phase-II Trial of Salvage Radiotherapy for Prostate Cancer In 4 Weeks vs. 2 Weeks	Weill Medical College of Cornell University	Genitourinary	Prostate Cancer	134	NCT04422132
MR-Linac for Head and Neck SBRT	Sunnybrook Health Sciences Centre	Head and Neck	Head and Neck Cancer	30	NCT04809792
Nano-SMART: Nanoparticles with MR Guided SBRT in Centrally Located Lung Tumors and Pancreatic Cancer	Dana–Farber Cancer Institute	Thorax	Non-small Cell Lung Cancer, Pancreatic Cancer	100	NCT04789486
Magnetic Resonance Guided Adaptive Stereotactic Body Radiotherapy for Lung Tumors in Ultra-central Location (MAGELLAN)	University Hospital Heidelberg	Thorax	Non-small Cell Lung Cancer, Metastatic tumors	38	NCT04925583
Study of LUNG Stereotactic Adaptive Ablative Radiotherapy (LUNG STAAR)	Baptist Health South Florida	Thorax	Non-small Cell Lung Cancer	60	NCT04917224
A Multicenter Phase-II Study of Stereotactic Radiotherapy for Centrally Located Lung Tumors (STRICT-LUNG STUDY) and Ultra-centrally Located Lung Tumors (STAR-LUNG STUDY)	Rigshospitalet, Denmark	Thorax	Primary Lung Cancer, Metastatic tumors	138	NCT05354596

## Data Availability

The data presented in this study are available in this article.
